# Supplementary material for: Prediction of the Risk of Malignancy of Adnexal Masses during Pregnancy Comparing Subjective Assessment and Non-Contrast MRI Score (NCMS) in Radiologists with Different Expertise
Source: Cancers (Basel). 2023 Oct 25;15(21):5138. doi: 10.3390/cancers15215138 (PMC10648807; doi:10.3390/cancers15215138)
Supplement: Supplementary file 1 [file cancers-15-05138-s001.zip › cancers-2592013-supplementary.pdf]

Supplementary Table S1. On Imaging Protocol sample.

Parameter	Axial T1-WI	Axial T2-WI	Axial DWI	Sagittal T2-WI	Coronal T2-WI	Axial T2-WI upper abdomen	Axial DWI upper abdomen
**Sequence**	3D FSPGR	FRFSE	EPI	FSE Propeller	FRFSE	FRFSE	EPI
**Imaging time**							
Echo time (msec)	Minimum/4.2	85	Minimum	85	85	84	Minimum
No. of signals acquired (NEX)	2	2	-	2	4	1	-
Repetition time (msec)	6.4	4,500	12,000	4,500	4,500	1,850	12,000
No. of sections	140	30	41	26	26	48	48
Receiver bandwidth (kHz)	90.91	31.25	250	41.67	41.67	41.67	250
Echo train length	-	26	-	15	26	17	-
**Imaging range**							
Field of view (mm)	350	240	320	240	240	460	460
Section thickness (mm)	1.60	4	4	4	4	5	5
Section spacing (mm)	1.06	0.5	0.5	0.4	0.5	1	1
Matrix size	320x224	384x256	128x128	384x256	384x256	256x256	128x128
Phase direction	R/L	R/L	A/P	S/I	R/L	R/L	A/P
b Value (sec/mm^2)	…	…	0-1000	…	…	…	0-1000

DW: diffusion weighted; EPI: echo-planar imaging; FSE: fast spin-echo; FSPGR: fast spoiled gradient echo; GRE: gradient-recalled echo; NPW: no phase wrap; SSFSE: single-shot FSE; WI: weighted-images.

Patient N°	Side	Maximal diameter (mm)	Hystolopathological result
1	Right	53	Decidualized endometrioma
2	Right	57	Serous Cystoadenoma
3	Left	77	Borderline Serous Papillary
4	Right	64	Decidualized endometrioma
5	Bilateral	97	Low-Grade Serous Adenocarcinoma
6	Left	71	Mature Cystic Teratoma
7	Left	104	Mature Cystic Teratoma
8	Left	131	Struma Ovarii
9	Left	70	Decidualized endometrioma
10	Right	64	Mature Cystic Teratoma
11	Right	72	Decidualized endometrioma
12	Bilateral	147	High-Grade Serous Adenocarcinoma
13	Left	44	Metastasis from sigmoid Adenocarcinoma
14	Left	103	Metastasis fromColon Adenocarcinoma
15	Right	136	Mucosecretor Adenocarcinoma
16	Right	38	Follow up
17	Right	160	Follow up
18	Right	83	Uterine Leiomyoma
19	Left	108	Mature Cystic Teratoma
20	Right	133	Borderline Mucinous

Supplementary Table S2. Characteristics of the masses for each patient included in the study (when bilateral, the measure was done on the biggest lesion).
